# Acute Endocarditis Following War Wounds

**Published:** 1919

**Authors:** 


**_Acute Endocarditis Following War Wounds._** By HOWARD T. KARSNER. *The Archives of Internal Medicine*, Sept. 15, 1918.

Endocarditis may accompany septicemia and pyemia. The treatment can be successfully modified by a consideration of the changes in myocardium and renal parenchyma which may permanently handicap the soldier in after life.

Of the fourteen cases showing acute valvular lesions, twelve were pure acute disease and two showed acute lesion superimposed on preceding chronic lesion.

The average duration of illness was eighteen days, as compared with an average of seventeen days in 42 septic cases without endocarditis.

All the cases were obviously either septicemia or pyemia, and the streptococcus pyogenes was the predominant organism.

The pure aortic cases showed a rather high age incident as compared with the other cases, excepting two patients who had acute and chronic lesions. This same group of men appeared to have been in active service somewhat longer than the other cases. Whereas acute aortic disease is common, it does not become as severe and extensive as disease of the other valves. Of considerable interest is the occurrence of two cases with valvular disease of the right side of the heart only. However, an examination of the data in these cases showed no explanation for their occurrence.

In only one case did the acute valvulitis appear to be of undoubted embolic origin: namely, the case of acute tricuspid disease, the lesion appearing as an ulcer in the middle of the valve flap, and associated with what appeared to be an abscess in the substance of the valve.

A case of pulmonic disease might have had a similar origin, for at the necropsy the lesion covered the ventricular surfaces of the leaflets and might have originated as emboli in the substance of the leaflets.

There were four cases of chronic valvular disease without acute lesion, two of which were septicemias of short duration (5 and 10 days). Of the septicemia cases, one showed a sclerosis of the valve rather than a true chronic inflammatory process, leaving one case of chronic valvulitis with septicemia and without acute lesion, as contrasted with two other cases with chronic valvulitis, pyemia, and a superimposed acute lesion.

The weight of the heart appears to be greater in men of long service and in older men, the difference being accentuated in men when greater age and longer service coincide. It is, therefore, tentatively concluded that long service in the army probably leads to an incresse in the weight of the heart, and that this change is more marked in men above the average age of this series of soldiers.

Averages were estimated of the age and term of service of 62 patients who showed sclerosis of the aorta, and 19 patients who failed to show it, and the resulting figures in each were 27 years of age and twenty-two months service. It is, therefore, unlikely that army service or the age of the soldier play any important part in the occurrence of arteriosclerosis, and that its cause must be sought elsewhere.

